# Impaired Temporal Processing of Tactile and Proprioceptive Stimuli in Cerebellar Degeneration

**DOI:** 10.1371/journal.pone.0078628

**Published:** 2013-11-11

**Authors:** Michele Tinazzi, Francesca Morgante, Alessia Peretti, Caterina Mariotti, Marta Panzeri, Mirta Fiorio, Alfonso Fasano

**Affiliations:** 1 Department of Neurological, Psychological, Morphological and Motor Sciences, University of Verona, Verona, Italy; 2 Dipartimento di Medicina Clinica e Sperimentale, University of Messina, Messina, Italy; 3 Unit of Genetics of Neurodegenerative and Metabolic Diseases, Fondazione IRCCS Istituto Neurologico ‘Carlo Besta’, Milano, Italy; 4 Movement Disorders Center, TWH, UHN, Division of Neurology, University of Toronto, Toronto, Ontario, Canada; University of Udine, Italy

## Abstract

Performance of timed motor sequences relies on the cerebellum and basal ganglia, which integrate proprioceptive information during the motor task and set internal timing mechanisms. Accordingly, these structures are also involved in other temporal processes, such as the discrimination of the different afferent information in the domain of time. In the present study we tested temporal processing of proprioceptive and tactile stimuli in 20 patients with neurodegenerative cerebellar ataxia and 20 age- and sex-matched healthy subjects. Tactile temporal discrimination threshold was defined as the value at which subjects recognized the two stimuli as asynchronous. Temporal discrimination movement threshold of the first dorsal interosseous and flexor carpi radialis was defined as the shortest interval between two paired electrical stimuli in which the subjects blindfolded perceived two separate index finger abductions and wrist flexions. Both tactile and movement temporal discrimination thresholds were higher in patients with cerebellar ataxia. No correlation was found with disease duration and severity. Our study demonstrates that temporal processing of tactile and proprioceptive stimuli is impaired in patients with cerebellar neurodegeneration and highlights the involvement of cerebellum in temporal processing of somatosensory stimuli of different type.

## Introduction

The performance of timed motor sequences relies on the cerebellum and basal ganglia, which integrate proprioceptive information during the motor task and set internal timing mechanisms. Specifically, the lateral cerebellum seems to be implicated mainly in the process of synchronization, whereas the basal ganglia seem to be implicated in internally paced timing mechanisms [1].

Tactile temporal discrimination threshold (TDT) and proprioceptive temporal discrimination movement threshold (TDMT) are psychophysical measures of temporal processing of tactile and proprioceptive stimuli [2], [3]. Functional magnetic resonance imaging (fMRI) studies demonstrated that several cortical and sub-cortical areas are involved in somatosensory temporal discrimination, among which basal ganglia and cerebellum [4]. Indeed, TDT has been considered as a marker of basal ganglia disease, as it has been consistently found abnormal in primary dystonia [2], [5], in non-manifesting DYT1 mutation carriers [6], in unaffected relatives of both familial and sporadic adult-onset dystonia patients [7] and in Parkinson’s disease [8]. TDMT has been found increased in Parkinson’s disease [9] and normal in focal hand, cervical and laryngeal dystonia [10], [11]. More recently, we investigated both measures in a sample of patients with essential tremor (ET) and found normal TDT values while TDMT was significantly higher than patients with dystonia or healthy controls [11]. Since ET specifically involves cerebellar and brainstem oscillating loops [12], TDT might be seen as a marker of basal ganglia impairment, whereas TDMT might be more closely related to cerebellar dysfunction. Indeed, proprioceptive sensory information involved in the TDMT is conveyed by the dorsal spinocerebellar tract and is processed through a distributed neural network in which the cerebellum and the parietal cortex play a prominent role [13]–[15]. Aim of the present study was to evaluate whether the cerebellum is involved in temporal processing of proprioceptive and tactile stimuli, by testing TDT and TDMT in patients with neurodegenerative cerebellar ataxia. If TDT and TDMT were related respectively to networks involving basal ganglia and cerebellum, we expect a selective impairment of TDMT in patients with cerebellar disease.

## Methods

### Subjects

We recruited 20 patients (5 women, 15 men, mean age, SD = 49.4±15.2 years) affected by neurodegenerative cerebellar ataxia (CA), from the outpatient clinic of the Neurology Institute “Carlo Besta” in Milan, Italy. All subjects gave their written informed consent before participation. The study was approved by the Department of Neurological, Psychological, Morphological and Motor Sciences, University of Verona. Inclusion criteria were the presence of a slowly progressive cerebellar syndrome without clinical evident involvement the motor (weakness, parkinsonism, dystonia, amyotrophy) and the sensory systems (such as somatosensory and visual systems). Eight patients had a positive family history compatible with an autosomal dominant disorder, and twelve cases were classified as sporadic cerebellar ataxia [16]. Genetic tests for SCA1, SCA2, SCA3, SCA6, SCA7, SCA17, DRPLA, SCA15, SCA28 were performed in all patients and a defined genetic diagnosis was achieved in five 5 of the familial cases (SCA28 = 2, SCA15 = 2, SCA6 = 1). In the sporadic cases and in three familial cases we did not identify a genetic cause. Secondary causes of cerebellar ataxia were excluded, such as metabolic alterations, vitamin E deficiency, and malabsorption. Severity of ataxia was evaluated by using the Scale for the Assessment and Rating of Ataxia (SARA) [17] which include 8 items, 3 of them being strictly related to the evaluation of the upper limbs coordination. As additional features, we demonstrated mild or moderate increase of deep tendon reflexes at lower limbs in 8 patients, lower limb spasticity in 1 case and ophthalmoparesis and slow saccades in the two SCA28 patients (Table 1). No patients had rigidity, bradykinesia, resting tremor or signs suggestive of parkinsonism or dystonia. Clinical evaluation of cognition was normal in all cases. Standard 1.5 Tesla brain Magnetic resonance imaging (MRI) demonstrated cerebellar atrophy in all patients. In none of the subjects atrophy of cerebral cortex, midbrain, pons and medulla or white matter lesions were identified. In the 2 subjects with SCA15 mild hypointensity of basal ganglia was evident. Further clinical and neuroimaging details are given in table 1. Neurophysiological investigations, including electromyography, peripheral nerve conduction, somatosensory and motor evoked potentials of the four limbs, and visual evoked potentials did not disclose any abnormality.

**Table 1:** Demographic and clinical features of patients with neurodegenerative cerebellar ataxia.

Patient	Age	Familial - Sporadic	Genotype	Disease Duration(Years)	SARA score	Cerebellar features	Additional Features	MRI Findings
1	60	Sporadic	Unknown	12	11	gait ataxia, mild dysarthria, limb dysmethria	DTR++	Cerebellar atrophy of vermis and hemispheres.
2	56	Sporadic	Unknown	35	10	gait ataxia, mild dysarthria, limb dysmethria, gaze evoked nistagmus, saccadic pursuit.	DTR+++, Bilateral EPR, mild increase of muscle tone at lower limbs	Atrophy of cerebellar vermis
3	50	Sporadic	Unknown	18	11	gait ataxia, mild dysarthria, limb dysmethria, gaze evoked nistagmus, saccadic pursuit.	DTR++	Cerebellar atrophy of vermis and hemispheres
4	63	Sporadic	Unknown	8	14	gait ataxia, mild dysarthria, limb dysmethria, gaze evoked nistagmus, saccadic pursuit.	//	Atrophy of cerebellar vermis
5	73	Sporadic	Unknown	36	31	gait ataxia, mild dysarthria, limb dysmethria, gaze evoked nistagmus	Slow saccades	Cerebellar atrophy of vermis and hemispheres
6	35	Sporadic	Unknown	8	8	gait ataxia, mild dysarthria, limb dysmethria, gaze evoked nistagmus	//	Cerebellar atrophy of vermis and hemispheres
7	68	Sporadic	Unknown	29	16	gait ataxia, mild dysarthria, limb dysmethria, gaze evoked nistagmus, decreased muscle tone	//	Cerebellar atrophy of vermis and hemispheres
8	22	Sporadic	Unknown	1	6.5	gait ataxia, mild dysarthria, limb dysmethria	//	Atrophy of cerebellar vermis
9	33	Sporadic	Unknown	13	10	gait ataxia, mild dysarthria, limb dysmethria	DTR +++	Cerebellar atrophy of vermis
10	46	Sporadic	Unknown	32	9	gait ataxia, mild dysarthria, limb dysmethria, decreased muscle tone	//	Cerebellar atrophy of vermis and hemispheres
11	47	Sporadic	Unknown	8	6.5	gait ataxia, mild dysarthria, limb dysmethria	//	Cerebellar atrophy of vermis and hemispheres
12	63	Sporadic	Unknown	6	5	gait ataxia, mild dysarthria, limb dysmethria	//	Very mild atrophy of cerebellar vermis.
13	49	Familial	Unknown	27	9	gait ataxia, no dysarthria,mild limb dysmethria	DTR ++	Cerebellar atrophy of vermis and hemispheres. Mild hyperintensity of cerebellar cortex.
14	27	Familial	Unknown	15	6	gait ataxia, mild dysarthria, limb dysmethria, gaze evoked nistagmus, saccadic pursuit.	//	Cerebellar atrophy of vermis and hemispheres.
15	51	Familial	Unknown	11	6	gait ataxia, mild dysarthria, limb dysmethria	//	Cerebellar atrophy of vermis and hemispheres
16	48	Familial	SCA28	30	13	gait ataxia, mild dysarthria, limb dysmethria, gaze evoked nistagmus	Ptosis, opthalmoparesis, slow saccades, DTR +++	Cerebellar atrophy of vermis and hemispheres
17	72	Familial	SCA28	52	11	gait ataxia, mild dysarthria, limb dysmethria, gaze evoked nistagmus	Ptosis, opthalmoparesis, slow saccades	Cerebellar atrophy of vermis and hemispheres
18	51	Familial	SCA15	10	11	gait ataxia, mild dysarthria, limb dysmethria,gaze evoked nistagmus	DTR +++	Atrophy of cerebellar vermis. Mild hypointensity of basal ganglia
19	23	Familial	SCA15	4	2	very mild gait ataxia, mild limb dysmethria, gaze evoked nistagmus	DTR +++	Atrophy of cerebellar vermis. Mild hypointensity of basal ganglia
20	52	Familial	SCA6	10	5	gait ataxia, mild limb dysmethria,severe dysarthria, gaze evoked nistagmus	//	Cerebellar atrophy of vermis and hemispheres

DTR = deep tendon reflexes (++ = brisk response;++ = very brisk response); EPR = extensor plantar response.

### Stimuli and Procedure

All participants were tested by a single neurologist expert in clinical neurophysiology (A.P.) in a single experimental session lasting about 2 hours. Each upper limb was tested separately, and the order of presentation including the stimuli procedure was counterbalanced across subjects. Verbal instructions about the experimental tasks were also provided before each testing session, as well as examples of single or double stimuli. To maintain subjects’ attention throughout the procedure and to disclose possible perseverative responses, catch trials (3 for each series) with inter-stimulus interval (ISI) of 0 ms were included during the ascending series for each procedure. Subjects were allowed to pause between each block.

#### Tactile temporal discrimination threshold (TDT)

Tactile TDT testing was conducted according to previous standardized protocols [18], [19]. For each hand, we repeated the measurements 4 times, thus obtaining 4 TDT values that were then averaged and entered in the data analysis. Paired tactile stimuli consisted of square-wave electrical pulses delivered by a constant current stimulator through surface skin electrodes (1 mm in diameter) attached with adhesive pads to the index finger of the right or the left hand. The anode was located 1.5 cm distally from the cathode. The intensity of tactile stimulation was determined for each subject, by delivering a series of stimuli at increasing intensity (from 1 mA). The minimal intensity at which electric stimuli were perceived in 10 out of 10 stimuli was used for testing and defined as the perceptual tactile threshold (PTT). Care was taken that stimuli induced no pain or discomfort. For each hand, combined stimuli were delivered in four separate blocks. Stimuli were delivered in pairs starting from simultaneous stimuli (ISI = 0 ms) and the ISI was progressively increased in 10 ms steps up to 300 ms. The value at which subject recognized the two tactile stimuli as sequential for at least 3 consecutive intervals was defined as TDT.

#### Temporal discrimination movement threshold (TDMT)

The first dorsal interosseous (FDI) and flexor carpi radialis (FCR) muscles were selectively stimulated with a procedure extensively described in previous studies [3], [10]. An insulated tungsten needle microelectrode (cathode) was inserted at the motor point (MP) of FDI and FCR muscles, previously localized. The MP corresponds to the position in a muscle at which threshold for evoked contraction is minimal and from which a clear twitch contraction is obtained with stimulation intensities of approximately 1 mA. The MP was first determined for each subject by surface stimulation through a probe electrode (2-mm diameter) in different positions over the muscle. During the experiment the microelectrode was inserted at the MP to provide stimulation. The anode was a surface electrode positioned 2–3 cm distally to the cathode. The stimulus (0.2 ms duration) was delivered with an intensity of stimulation ranging from 1 to 2 mA and produced a non-painful twitch of the FDI muscle (causing an index finger abduction) and of the FCR muscle (causing a wrist flexion), without inducing radiating cutaneous paresthesias or sharp sensations. The minimal intensity at which subjects perceived finger abduction and wrist flexion movements in 10 out of 10 stimuli was used for testing and defined as the perceptual proprioceptive threshold (PPT). For each muscle, combined stimuli were delivered in four separate blocks. Before starting the task, subjects were trained to maintain the FDI and FCR muscles relaxed helped by EMG auditory feedback. When subjects were able to maintain the muscles relaxed, the testing session started. During the testing session, the subjects were blindfolded and wore ear-plugs to prevent visual and auditory feedback. Stimuli were delivered in pairs starting from simultaneous stimuli (ISI = 0 ms) and ISIs were progressively increased in 10 ms steps up to 300 ms. We defined TDMT as the shortest interval elapsing between two paired electrical stimuli in which the subjects blindfolded perceived two separate index finger abductions (in response to FDI stimulation) and wrist flexions (in response to FCR stimulation) for at least three consecutive intervals. For each muscle, the values obtained from 4 measurements were averaged and entered in the data analysis.

### Statistical Analysis

Two-sample t-test and Fisher’s exact test were used to explore between-groups differences in demographical variables. Separate repeated measures analyses of variance (R-ANOVAs) were employed to compare groups for each variable (PT, PPT, TDT, TDMT). R-ANOVA for PT and TDT had one between-subjects factor: “Group” (2 levels: CA, HC) and one within-subjects factor: “Side” (2 levels: right, left). R-ANOVA for PPT and TDMT had one between-subjects factor: “Group” (2 levels: CA, HC) and two within-subjects factors: “Side” (2 levels: right, left) and “Muscle” (2 levels: FDI, FCR). Conditional on significant F-value, post-hoc comparisons were performed by means of two-samples t-tests with Bonferroni’s correction where necessary. Correlations between TDMT or TDT abnormalities with disease severity (by SARA) and duration were explored by Spearman rank correlation. Significance level was set at P<0.05. Unless otherwise stated, data are given as mean ± standard error (SE).

## Results

No differences were found between CA and HC for age (*p* = 0.3, independent-sample t-test) and gender distribution (*p* = 1, Fisher’s exact test). PT and PPT did not significantly differ between CA patients and HC for both sides (Table 2).

**Table 2:** Perceptual tactile and proprioceptive thresholds, TDT and TDMT in cerebellar ataxia and healthy subjects.

	Cerebellar Ataxia	Controls	P - value	t-value
PTT (right hand)(mA)	8.2±0.8	7.9±0.8	0.8	0.3
PTT (left hand)(mA)	8.0±0.7	8.4±0.9	0.7	−0.4
PPT – right FDI(mA)	1.69±0.05	1.61±0.06	0.3	1.02
PPT – left FDI(mA)	1.7±0.04	1.62±0.06	0.2	1.3
PPT – right FCR(mA)	1.76±0.04	1.72±0.05	0.5	0.6
PPT – left FCR(mA)	1.75±0.07	1.71±0.04	0.6	0.5
TDT (right)(ms)	93.7±7.3	58.7±2.04	<0.0001*	4.6
TDT (left)(ms)	92.6±6.7	60.7±1.9	<0.0001*	4.5
TDMT FDI (right)(ms)	108.3±3.7	92.7±1.3	0.0004*	3.9
TDMT FDI (left)(ms)	106.7±3.7	93.5±0.9	0.001*	3.5
TDMT FCR (right)(ms)	120.4±3.5	105.6±0.9	0.0003*	4.0
TDMT FCR (left)(ms)	119.0±3.4	106.1±1.1	0.001*	3.5

PTT = perceptual tactile threshold; PPT = perceptual proprioceptive threshold; TDT = tactile temporal discrimination threshold; TDMT = proprioceptive temporal discrimination movement thresholds; FDI = first dorsal interosseous; FCR = flexor carpi radialis.

* Significant with Bonferroni’s correction.

All neurophysiological data are given as mean ±SE.

**Figure 1:**
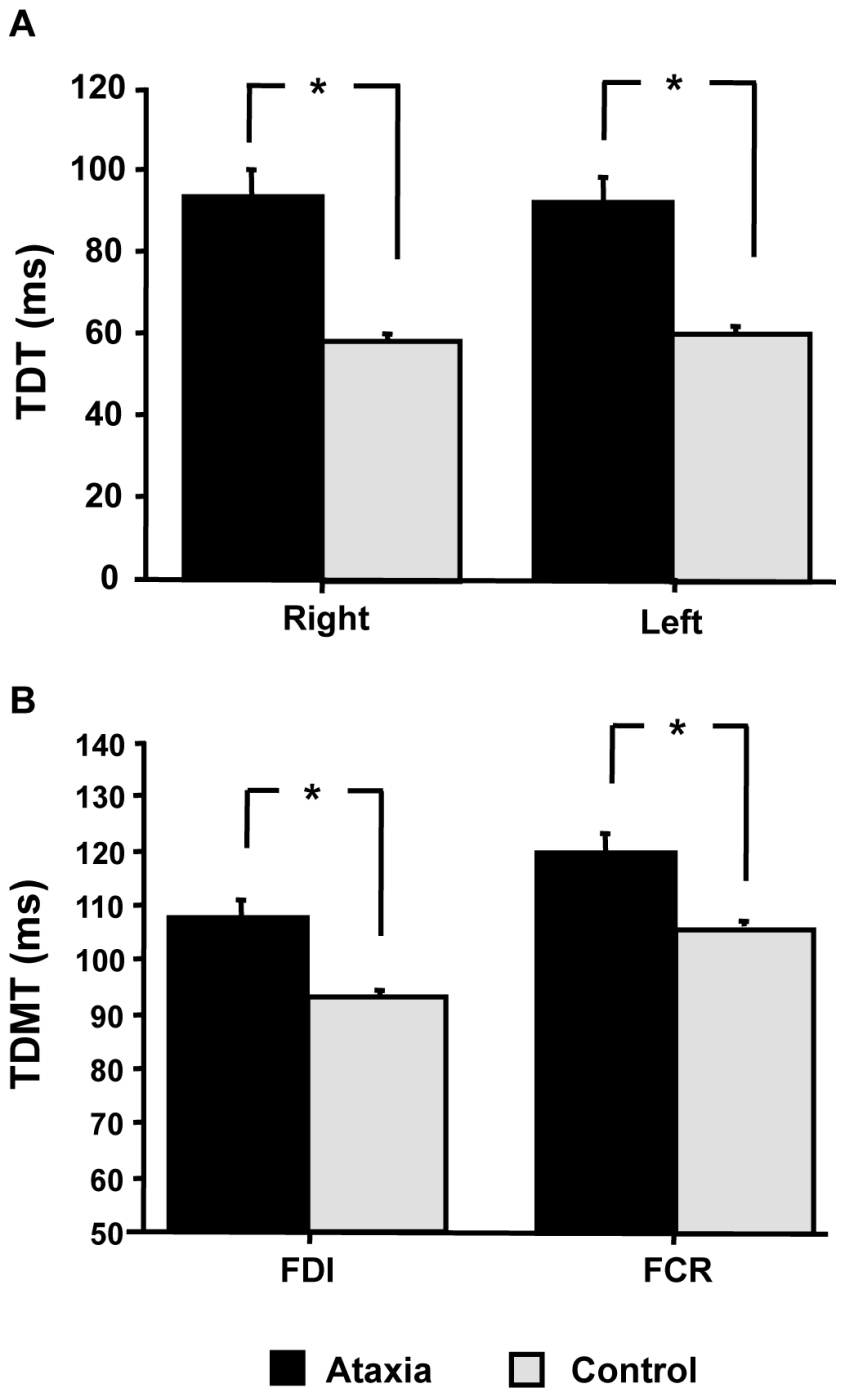
Temporal processing of tactile and proprioceptive stimuli in cerebellar degeneration. Increased tactile temporal discrimination threshold (TDT) (panel A) and temporal discrimination movement threshold (TDMT) (panel B) in patients with cerebellar degeneration compared to control subjects. For TDMT the average value of right and left TDMT is shown. *P<0.001.

For TDT, R-ANOVA disclosed a main effect of Group *(*F_1,38_ *= 22.2; p*<0.0001), due to higher TDT in CA patients compared to control subjects. Side (F_1,38_ = 0.06; *p* = 0.8) and Side by Group interactions were not significant (F_1,38_ = 0.8; *p* = 0.4) (Figure 1, panel A). For TDMT, R-ANOVA identified a main effect of Group (F_1,76_ = 30.9, *p*<0.0001), due to higher TDMT in CA than in HC (Figure 1, panel B). The factor Muscle was also significant (F_1,76_ = 23.9, *p*<0.0001), with higher TDMT in FCR than in FDI muscle in both groups. Side (F_1,76_ = 0.3, *p* = 0.6), Side by Group interaction (F_1,76_ = 1.7, *p* = 0.2) and Side by Muscle by Group interaction were not significant (F_1,76_ = 0.02, *p* = 0.9). Further statistical details are reported in the Table 2.

In CA patients, Spearman rank correlation coefficients disclosed no significant correlation between TDMT or TDT abnormalities and disease duration (r = −0.1, p = 0.39 for TDT; r = 0.08, p = 0.7 for TDMT- FDI; r = −0.03, p = 0.9 for TDMT-FCR) or severity, as evaluated with SARA (r = −0.09, p = 0.7 for TDT; r = −0.08, p = 0.7 for TDMT-FDI; r = −0.1, p = 0.6 for TDMT-FCR).

## Discussion

Our study showed that temporal processing of tactile and proprioceptive stimuli, evaluated by a psychophysical discriminative paradigm, is impaired in patients with neurodegenerative cerebellar ataxia. Abnormal temporal processing did not correlate with disease duration or severity. This might be explained by the lack of patients with short disease duration, as most of the subjects had a long disease duration (≥10 years in 70% of them). Alternatively, together with the lack of correlation with disease severity, we can speculate that impaired temporal discrimination is an abnormality not related to motor disability but rather reflecting dysfunction of the cerebellar pathways.

### Cerebellum and Temporal Discrimination of Proprioceptive Stimuli

The novel finding of our study is the increase of TDMT in patients with cerebellar degeneration. TDMT is a psychophysical technique measuring the temporal aspects of kinaesthesia [3]. TDMT in normal subjects and patients with cerebellar degeneration was higher in the flexor carpi radialis than in fist dorsal interosseous. This finding might be due to the highest density of muscle spindle in intrinsic muscles of the hands compared to the forearm muscles, thus conveying a larger amount of proprioceptive inputs [20].

Kinaestesia is an aspect of proprioceptive sensation with refers to the ability to sense the position and movement of a body part [14]. Previous studies in patients with cerebellar lesions reported conflicting results on the role of cerebellum in kinaestesia. The ability to perceive kinaestetic stimuli was impaired in patients with cerebellar degeneration, especially on tasks testing for duration and velocity perception [21]. However, another study examining the elbow position sense found a worse performance in patients with PD compared to those with spinocerebellar ataxia type 6 [22]. These discrepancies might arise from having studied different aspects of kinaestesia; indeed, recent evidence suggest that sense of position and sense of movement are separate senses and their central processing involves different networks [14]. Specifically, the sense of movement recognizes a cerebellar elaboration [14] and the left cerebellum appears to be critical in predicting the sensory consequences of an action, comparing visual and kinaesthetic inputs during movements [23].

Our previous studies demonstrated that TMDT – which explores the sense of movement in the domain of time – mainly involve activation of muscle afferent fibres [3] and suggested the involvement of cerebellum, as increased TDMT was found in patients with ET [11]. Accordingly, a functional MRI study in healthy subjects showed that contralateral primary and secondary somatosensory areas and the cerebellum were mostly activated during electrically induced ankle dorsiflexion than at rest [24].

In conclusion, increased TDMT in patients with cerebellar degeneration support the role of cerebellum in updating the somatosensory cortex with proprioceptive inputs coming from muscle afferents during performance of movements; the alteration of temporal proprioceptive discrimination in patients with cerebellar degeneration support the role of cerebellum in performing computations of time differences, for all sensory modalities [25].

### Cerebellum and Temporal Discrimination of Tactile Stimuli

There are few possible interpretations about the increase of TDT in the patients with cerebellar disease. First, subtle abnormalities of peripheral sensory nerves might have influenced our results; however, this is unlikely as nerve conduction studies and evoked potentials did not disclose any significant abnormality in the sensory system in none of the patients. Moreover, perceptual tactile and proprioceptive thresholds were similar among patients and controls. As second hypothesis, presence of extra-cerebellar pathology in the basal ganglia might have determined higher tactile TDT; again, none of subjects presented with dystonic or parkinsonian features, which are reported to be associated with increased TDT [5], [8]. However, a subtle not clinically evident involvement of the basal ganglia cannot be ruled out, especially taking into account the widespread degenerative processes seen in neurodegenerative CA.

Finally, impaired temporal processing of tactile stimuli might be caused by cerebellar degeneration itself. This finding was unexpected, as discriminative threshold for tactile stimuli has been considered to engage mainly basal ganglia processing; indeed, increased tactile TDT has been consistently reported in PD [8] and dystonia [5], [11], [18], [26]. Moreover, a voxel-based morphometry study demonstrated a bilateral increase in putaminal grey matter only in those unaffected relatives of adult-onset dystonia patients in whom tactile TDT was increased [7], further supporting the hypothesis that TDT mainly involves basal ganglia processing. However, functional neuroimaging studies showed that, beyond primary sensory areas and basal ganglia, several cortical and sub-cortical areas such as the pre-supplementary motor area, the anterior cingulate cortex and the cerebellum itself are involved during a tactile TDT task [4], [27], [28]. In keeping with these neuroimaging data, TDT was recently found increased in a small sample of patients with cerebellar atrophy [29]; our study confirmed and expanded these results in a larger sample of patients with cerebellar degeneration, including also patients with a defined genetic diagnosis.

Against the hypothesis of cerebellar involvement in time processing of tactile stimuli, a recent study found that continuous theta-burst transcranial magnetic stimulation over of the lateral cerebellum left TDT unchanged [30]. Furthermore, we recently found no impairment of TDT in patients with ET [11], a disorder thought to originate within cerebellar and brainstem oscillating loops [12]. The reason why TDT is not affected in ET patients while it is impaired in patients with cerebellar degeneration, in spite of a common anatomo-functional substrate, is currently unknown. A possible explanation is the different severity or level of cerebellar involvement: though the dysfunctional rather than degenerative nature of cerebellar involvement in ET is currently debated [31], patients with cerebellar ataxia bear widespread changes of cerebellar cortical layers and deep nuclei, thus including basal ganglia.

Overall, our data of increased tactile TDT in patients with cerebellar degeneration highlight the contribution of cerebellum in temporal discrimination of tactile stimuli and challenges the view that this psychophysical paradigm might represent a marker of basal ganglia impairment.

### Contribution of the Cerebellum and Basal Ganglia to Sensory Processing

Accurate performance of voluntary movements relies on correct integration of sensory inputs which provide information on velocity and position, as well on visual and auditory feedback.

Sensory information necessary to provide adequate motor control is conveyed both in the basal ganglia and the cerebellum. Basal ganglia function is to select between competing options in order to produce either goal-directed or habitual behaviours, which in turn depend on segregated functional territories [32]. Sensory inputs into the striatum come from the midline thalamic nuclei an their role might be to focus attention in order to recognize the information to be perceived and suppress unwanted confusing sensory information [33].

On the other side, the cerebellum receives massive sensory inputs as demonstrated by functional neuroimaging studies disclosing cerebellar activation during cutaneous, kinaesthetic, visual or auditory stimulation [34]–[37].

Our data of abnormal TDT and TDMT in cerebellar neurodegeneration are consistent with the role of the cerebellum in timing processing of stimuli of different type and specifically in timed event prediction. Impairment on perceptual tasks that require precise timing and poor acuity on a time discrimination task has been reported in patients with cerebellar lesions [38], [39]; in addition, neurophysiological and neuroimaging studies suggest that the cerebellum cooperate with basal ganglia to regulate timing of movement and perception [25], [40], [41]. At this regards, the cerebellum might modulate the sensory cortex, by evaluating the predictability of incoming somatosensory sensory stimuli [42]. This hypothesis is supported by a somatosensory evoked potentials study in patients with lateralized cerebellar which showed that cerebellar lesions impair intracortical processing of somatosensory stimuli [43].

### Implications of the Results

Similarly to PD patients and differently from subjects with dystonia (table 3), our data demonstrate that patients with cerebellar neurodegeneration do not exhibit a modality-selective abnormality in temporal discrimination.

**Table 3:** Synopsis of the available information on TDT and TDMT in disorders of the central nervous system.

*Condition*	*TDT*	*TDMT*	*Reference Number*
Parkinson’s disease	Abnormal	Abnormal	8,9
Essential Tremor	Normal	Abnormal	11
Dystonia	Abnormal	Normal	2,5–7, 11, 17–19
Cerebellar ataxia	Abnormal	Abnormal	This study

TDT: temporal discrimination threshold; TDMT: temporal discrimination movement thresholds (TDMT).

There are few possible explanations for this finding: first, it should be also considered that the pattern of neurodegeneration in PD is not limited to the basal ganglia, but can functionally affect interconnected areas such as the cerebellum itself [44]; alternatively, we can speculate that the impairment of temporal discrimination of tactile and proprioceptive stimuli might be partly caused by a specific impairment of executive/attentive resources which was demonstrated in both these conditions [45]–[47]. However, we were not able to systematically assess our cohort by means of an extensive neuropsychological battery for attentive/executive impairments; in addition none of the previous studies evaluating TDT or TDMT have ever correlated this alteration to impairment of executive function or attention. Regarding attention, we tried to minimize this bias, by delivering catch trials (3 for each series) with inter-stimulus interval of 0 ms as already described in previous studies [5], [48].

Finally, the alteration of TDT in patients with cerebellar degeneration lead us to reconsider the meaning of higher TDT in dystonia [5], [11], [18], [26]. So far, impaired somatosensory temporal processing in dystonia has been considered an endophenotype [6] of this disease arising from a dysfunction of basal ganglia [7]. However, on the basis of the results of the present study, we should consider that some of the abnormalities of sensory processing in dystonia might be also accounted for cerebellar involvement, as suggested by recent studies showing functional abnormalities in the cerebello-thalamo-cortical pathway in dystonia [49]. In conclusion, our data of impaired tactile TDT and TDMT in cerebellar ataxia, support the notion that cerebellum is involved in temporal processing of somatosensory and kinaestetic stimuli. The meaning of TDT and TDMT alteration diseases involving the basal ganglia and the cerebellum needs to be better disclosed, by designing studies correlating TDT and TDMT with clinical (bradykinesia, dystonia, ataxia) and cognitive (executive function/attention) features.
